# Identification and predictive machine learning model construction of gut microbiota associated with carcinoembryonic antigens in colorectal cancer

**DOI:** 10.1128/msphere.00454-25

**Published:** 2025-09-17

**Authors:** Yongzhi Wu, Zigui Huang, Yongqi Huang, Chuanbin Chen, Mingjian Qin, Zhen Wang, Fuhai He, Shenghai Liu, Rumao Zhong, Jun Liu, Chenyan Long, Jungang Liu, Xiaoliang Huang

**Affiliations:** 1 Division of Colorectal & Anal Surgery, Department of Gastrointestinal Surgery, Guangxi Medical University Cancer Hospital 117981, Nanning, The People’s Republic of China; 2 Guangxi Key Laboratory of Basic and Translational Research on Colorectal Cancer, Guangxi, The People’s Republic of China; University at Buffalo-Downtown Campus, Buffalo, New York, USA

**Keywords:** colorectal cancer, carcinoembryonic antigen, intestinal microbiology, 16S rRNA, machine learning

## Abstract

Carcinoembryonic antigen (CEA) is a critical colorectal cancer (CRC) biomarker, but its mechanistic link to gut microbiota remains unclear. This study characterized gut microbiota differences between high-CEA (H-CEA) and low-CEA (L-CEA) CRC patients and explored their associations with host immunity and tumor progression mechanisms. Stool samples from 187 CRC patients were subjected to 16S rRNA sequencing, identifying 30 differentially abundant bacteria using LEfSe analysis. *Ruminococcus callidus* was significantly enriched in H-CEA patients. Transcriptome sequencing of tumor tissues from 25 patients revealed distinct immune micro-environments: H-CEA patients showed elevated resting memory CD4^+^ T cells, while L-CEA patients showed increased T follicular helper cells. Functional enrichment analysis identified differential GO terms (26 in L-CEA; 31 in H-CEA) and KEGG pathways (three in H-CEA). *R. callidus* correlated positively with mast cell infiltration, CXCL1 chemokine, and long-chain fatty acid upregulation. The area under the curve (AUC) values of the subjects in the training set for the RF and XGBoost models constructed based on differential gut microbiota for predicting high and low CEA levels were 0.969 and 0.815, respectively, and the AUC for the test set were 0.715 and 0.639. These findings demonstrate that CEA-level-specific gut microbiota dysbiosis modulates CRC progression through immune micro-environment alterations and related biological pathway regulation. Gut microbiota, as a noninvasive biomarker, can be used to construct an effective machine learning (ML) model for predicting blood CEA levels.

**IMPORTANCE:**

This study reveals *R. callidus* as a key gut microbiota species enriched in CRC patients with high CEA levels, demonstrating its novel pro-tumor associations through positive correlations with mast cell infiltration and CXCL1 chemokine and upregulation of long-chain fatty acid metabolism. Concurrently, we identify distinct immune micro-environments: elevated resting memory CD4+ T cells in high-CEA patients versus increased T follicular helper cells in low-CEA cohorts. Critically, by leveraging 30 differential microbial features, we develop ML models for noninvasive prediction of CEA levels. These findings establish gut microbiota as both a mechanistic mediator of CEA-driven CRC progression and a foundation for microbiome-based diagnostic tools.

## INTRODUCTION

The latest epidemiological data showed that the incidence of CRC ranks steadily among the top three malignant tumors, with nearly 2 million new cases per year; the mortality rate ranks second among cancer-related deaths, with about 1 million deaths per year (1). In this context, early screening and accurate diagnosis are of key importance to improve clinical prognosis, and CEA is both a serologic indicator for adjuvant to diagnosis of various cancers and a prognostic biomarker for dynamic monitoring of disease regression (2, 3).

In the clinical management of CRC, serum CEA concentration has a clear clinical threshold significance. A CEA level >3–5 ng/mL suggests abnormally elevated, >10 ng/mL is highly associated with malignant lesions, and a level >20 ng/mL requires vigilance for metastatic risk (3–5). First, CEA directly binds to TRAIL-R2 (DR5), which significantly enhances metastatic cell survival by reducing hypoxia through exogenous pathways (6). Second, CEA interoperates with the hnRNP M4 on the surface of Kupffer cells, inducing the release of inflammatory mediators such as IL-1β and TNF-α (7), upregulating the expression of endothelial adhesion molecules in hepatic sinusoids, and accelerating CRC cell-specific anchoring and liver micro-environment colonization. Furthermore, CEA may synergize with inflammatory factors to activate the NF-κB pathway, trigger the release of chemokine cascades (8), and recruit immune cells to build an immune-suppressive micro-environment and promote tumor immune escape. It is worth noting that although CEA is widely used in early CRC screening, its expression lacks tissue specificit, and its causal association with CRC development has not been fully elucidated. As a key regulator of CRC progression, the interaction network between gut microbiota and CEA remains to be resolved (9), which provides an important direction for in-depth mechanistic studies.

In recent years, technological innovations in gut microbiome research, including 16S rRNA gene sequencing, macro-genomics, metabolomics, and transcriptomics analyses (10–12) have greatly expanded the depth of knowledge about the function of the gut microbiota. Joint multi-omics studies have confirmed the involvement of specific microbiota in the cancer process through direct metabolic intervention or immunomodulation (13), with *Fusobacterium nucleatum* (12), enterotoxigenic *Bacteroides fragilis*, and *pks^+^ Escherichia coli* being clearly identified as the high-risk causative agents of CRC. Notably, the abnormal accumulation of fatty acids in the intestines of CRC patients forms a vicious circle with dysbiosis. On the one hand, tumor cells consume large amounts of fatty acids to meet their proliferative demands, leading to the increased abundance of sulfate-reducing bacteria and pro-inflammatory microbiota in the intestinal microenvironment (14); on the other hand, high saturated fatty acid intake significantly reduces the expression of tight junction-related proteins claudin-1 and occludin by upregulating the abundance of sulfate-reducing bacteria (15), increasing the intestinal mucosal permeability with the plasma lipopolysaccharide binding protein levels, which in turn activates chronic inflammatory pathways. Changes in the gut microbiota also lead to a decrease in the production of short-chain fatty acids (SCFAs) in the lumen of the colon, and the absence of SCFAs not only weakens the intestinal epithelial barrier function but also disrupts immune homeostasis through the mechanisms of regulating the differentiation of T cells and the inhibition of histone deacetylase (16). In conclusion, the metabolic-microbial axis composed of fatty acid metabolism disorders and ecological imbalance of the microbiota has become a core regulatory network that cannot be ignored in the pathologic process of CRC.

In this study, 187 CRC patients were included, preoperative stool samples were collected for 16S rRNA gene sequencing, and 25 paired tumor tissues were selected for transcriptomic sequencing. By integrating multi-omics data, we systematically analyzed the characteristics of the gut microbiota of patients with different serum CEA levels and their interactions with the tumor immune micro-environment and then constructed RF and XGBoost prediction models based on the characteristic microbial markers, which realized the non-invasive assessment of serum CEA concentration.

## RESULTS

### Clinical data and statistical characteristics of CRC patients enrolled in the study

In this study, 198 stool samples were collected from CRC patients who met the inclusion criteria for 16S rRNA sequencing analysis, and 187 subjects with complete CEA data were selected. The subjects were divided into two groups according to CEA levels: 93 in the H-CEA group and 94 in the L-CEA group. The samples were collected using a sequential enrollment strategy to truly reflect the heterogeneity of the clinical population. As shown in Table 1, there were no significant differences (*P* > 0.05) between the two groups of subjects in several baseline characteristics, including age (*P* = 0.844), gender (*P* = 0.341), BMI (*P* = 0.453), tumor localization (*P* = 0.317), tumor volume (*P* = 0.431), perineural invasion (*P* = 0.625), and lymph-vascular invasion (*P* = 0.980). However, there was a statistical difference in the feature of tumor TNM stage (*P* = 0. 023). Overall, the natural distribution of subjects in the two groups was balanced in terms of key potential confounders, which provided a valid base of support for subsequent microbiome analysis.

**TABLE 1:** Demographic and clinical characteristics of CRC patients stratified by high and low CEA^*a*^

Characteristic		L-CEA (*n* = 94)	H-CEA (*n* = 93)	*P* value	Test
Age (years, mean ± SD)		58.41 ± 11.51	58.09 ± 11.37	0.844	*t*-test
Age (%)				0.702	Pearson χ^2^
<60		53 (56.4)	56 (60.2)		
≥60		41 (43.6)	37 (39.8)		
Gender (%)				0.341	Pearson χ^2^
Male		59 (62.8)	51 (54.8)		
Female		35 (37.2)	42 (45.2)		
BMI (%)				0.453	Pearson χ^2^
<24.0		63 (67.0)	68 (73.1)		
≥24.0		31 (33.0)	25 (26.9)		
Tumor localization (%)				0.317	Pearson χ^2^
Left colon		20 (21.3)	26 (28.0)		
Right colon		26 (27.7)	16 (17.2)		
Rectum		47 (50.0)	49 (52.7)		
Transverse colon		1 (1.0)	2 (2.1)		
Tumor volume (cm^3^, mean ± SD)		22.26 ± 53.81	17.03 ± 26.90	0.431	*t*-test
TNM stage (%)				0.023	Pearson χ^2^
Early (0 ~ 2)		39 (41.5)	23 (24.7)		
Advanced (3 ~ 4)		55 (58.5)	70 (75.3)		
Perineural invasion (%)				0.625	Pearson χ^2^
No		35 (37.2)	35 (37.6)		
Yes		36 (38.3)	27 (29.0)		
Lymph-vascular invasion (%)				0.980	Pearson χ^2^
No		53 (56.4)	48 (51.6)		
Yes		17 (18.1)	14 (15.1)		

^*a*^ *P* values < 0.05 were statistically significant.

### Comparison of microbiome diversity in the L-CEA and H-CEA groups

**Fig 1:**
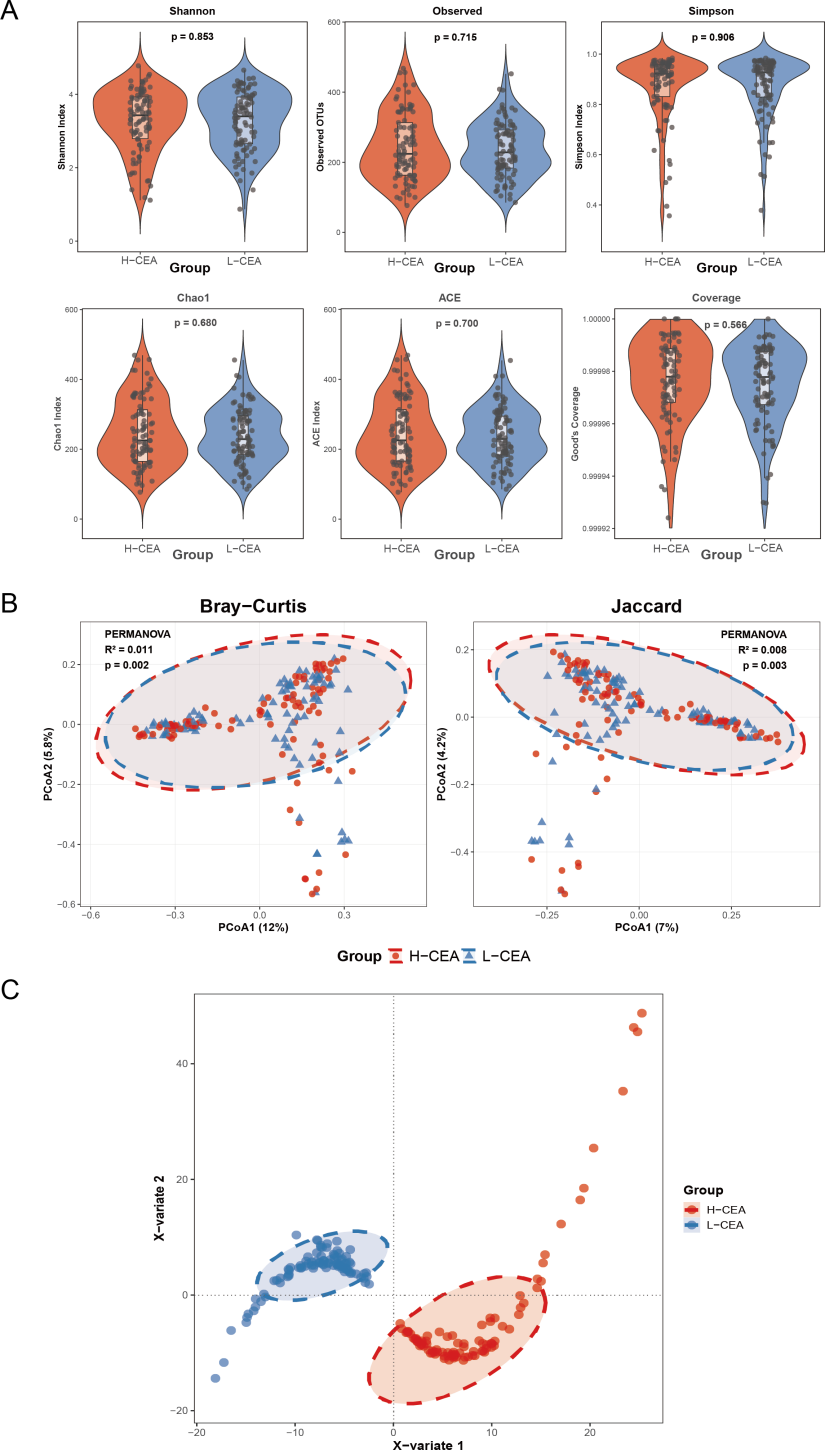
Comparison of the microbiome diversity index of CRC patients in H-CEA and L-CEA (**A**) Comparison of the α-diversity index of gut microbiota between H-CEA and L-CEA of the CRC patient group. Species diversity differences between two groups were analyzed using Wilcoxon rank-sum tests, considering *P* < 0.05 as statistically significant. Significance was further verified by applying the Bonferroni correction for multiple hypothesis testing (FDR-adjusted *P*-values). Six α-diversity indices are presented in the figure, with group assignment on the *x*-axis and diversity index values on the *y*-axis. (**B**) Comparison of the β-diversity index of gut microbiota between H-CEA and L-CEA. Principal coordinate analysis (PCoA) plots based on Bray-Curtis and Jaccard distances show the separation of gut microbial community structure between the two groups. Ellipses represent 95% confidence intervals, red circles represent the H-CEA group, and blue triangles represent the L-CEA group. PERMANOVA showed significant differences in microbial community composition between the two groups (Bray-Curtis: *R*² =0. 011, *P* = 0. 002; Jaccard: *R*² =0. 008, *P* = 0. 003). (**C**) X-variable clustering plots between H-CEA and L-CEA. Red color represents the H-CEA group, and blue color represents the L-CEA group. The X variable 1 and X variable 2 in vertical and horizontal coordinates represent the main clustering feature dimensions, respectively.

In order to investigate the potential differences in the diversity of the gut microbiome between the two groups, we comprehensively analyzed the diversity and structure of the microbial communities using 16S rRNA sequencing. Figure 1A presents the statistical results of the six α-diversity indices, and all *P*-values exceeded 0.05, indicating that there were no significant differences between the two groups in terms of species richness, community homogeneity, and sequencing depth of the gut microbiota. Figure 1B shows the results of β-diversity analyses by principal coordinate analysis (PCoA), and the analyses based on Bray-Curtis distance (*R*²= 0. 011, *P* = 0. 002) and Jaccard distance (*R*²= 0. 008, *P* = 0. 003) revealed significant differences between the two groups of samples, suggesting that the levels of CEA may be associated with the structure of the gut microbial community. Further PLS-DA (Fig. 1C) successfully distinguished the L-CEA group from the H-CEA group. These findings highlight that while α-diversity remains homogeneous, β-diversity, particularly species composition, is a key feature in distinguishing the two microbiomes.

### Exploring differential gut bacteria associated with CEA

**Fig 2:**
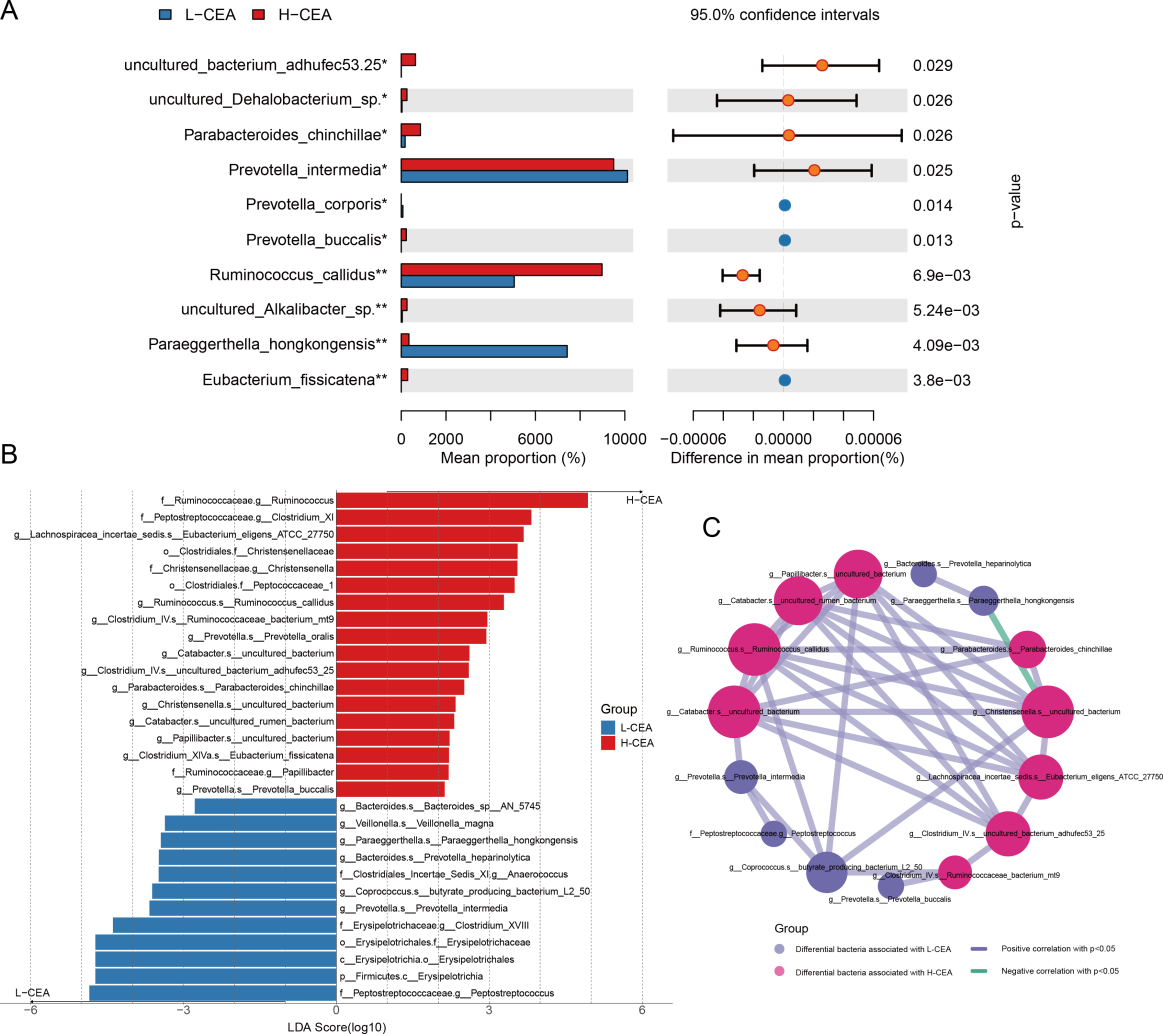
Differential analysis of gut microbial communities in different CEA level groups. (**A**) Comparison of absolute abundance of species taxon in two groups of patients. Absolute abundance of gut microbiota (range: 0 to 80,000) is plotted on the *y*-axis against distinct bacterial taxa on the *x*-axis. For each taxon, paired bars depict mean abundance in the H-CEA and L-CEA groups, accompanied by error bars signifying standard deviation or confidence interval. Asterisks denote statistically significant inter-group differences: * (*P* < 0.05), ** (*P* < 0.01), and *** (*P* < 0.001). (**B**) LDA bar graph based on 16S rRNA gene sequencing. Bar color denotes group affiliation. The *x*-axis shows the log10-transformed LDA score, while the *y*-axis lists significantly enriched species within each group. Bar length corresponds to the LDA score magnitude. (**C**) Network analysis of CEA-associated differential gut microbiota correlations. Nodes represent species, colored by group. Node size scales with connectivity (number of edges). Edges signify significant correlations (Spearman’s rho, *P* < 0.05 after FDR correction): blue lines for negative (*r* < 0) and yellow lines for positive (*r* > 0) associations. Edge thickness indicates correlation strength.

In order to gain a deeper understanding of the differences in the composition of the gut bacteria and the potential interactions between the two groups of patients, we used a combination of LEfSe analysis and microbial correlation network analysis. Figure 2A illustrates the top ten bacterial species with significant abundance differences between the two groups of patients, with *Ruminococcus_callidus*, *Paraeggerthella_hongkongensis*, and *Eubacterium_fissicatena* showing particularly significant differences. Through LEfSe analysis (log10-transformed), shown in Fig. 2B and Table S1, we further revealed microbial taxonomic units that were significantly different between the two groups. The LDA scores on the horizontal axis of the graph reflect the enrichment of these taxonomic units in the corresponding group, with higher scores indicating higher enrichment in that group. It was found that a total of 30 taxa with different taxonomic levels showed statistically significant differences in abundance between the two groups, among which 18 taxa were significantly more abundant in the H-CEA group than in the L-CEA group. Specifically, *Ruminococcus_callidus* and *Eubacterium_fissicatena* were significantly enriched in the H-CEA group, whereas *Prevotella_intermedia* and *Prevotella_heparinolytica* were significantly enriched in the L-CEA group.

To further reveal the interactions within the microbial communities of the two groups, we constructed a network diagram of the correlation of intestinal dominant bacteria with species as the level of categorization (Fig. 2C). In the network diagram, red nodes represent bacteria significantly enriched in the H-CEA group, and blue nodes represent bacteria significantly enriched in the L-CEA group. The blue connecting line indicates a positive correlation, and the green connecting line indicates a negative correlation. The results of the analysis showed that there were complex interconnections and interactions between the dominant bacteria in the two groups, suggesting that there may be potential competitive and synergistic relationships between the two groups of dominant bacteria.

### Functional prediction of gut microbiota in H-CEA and L-CEA groups

**Fig 3:**
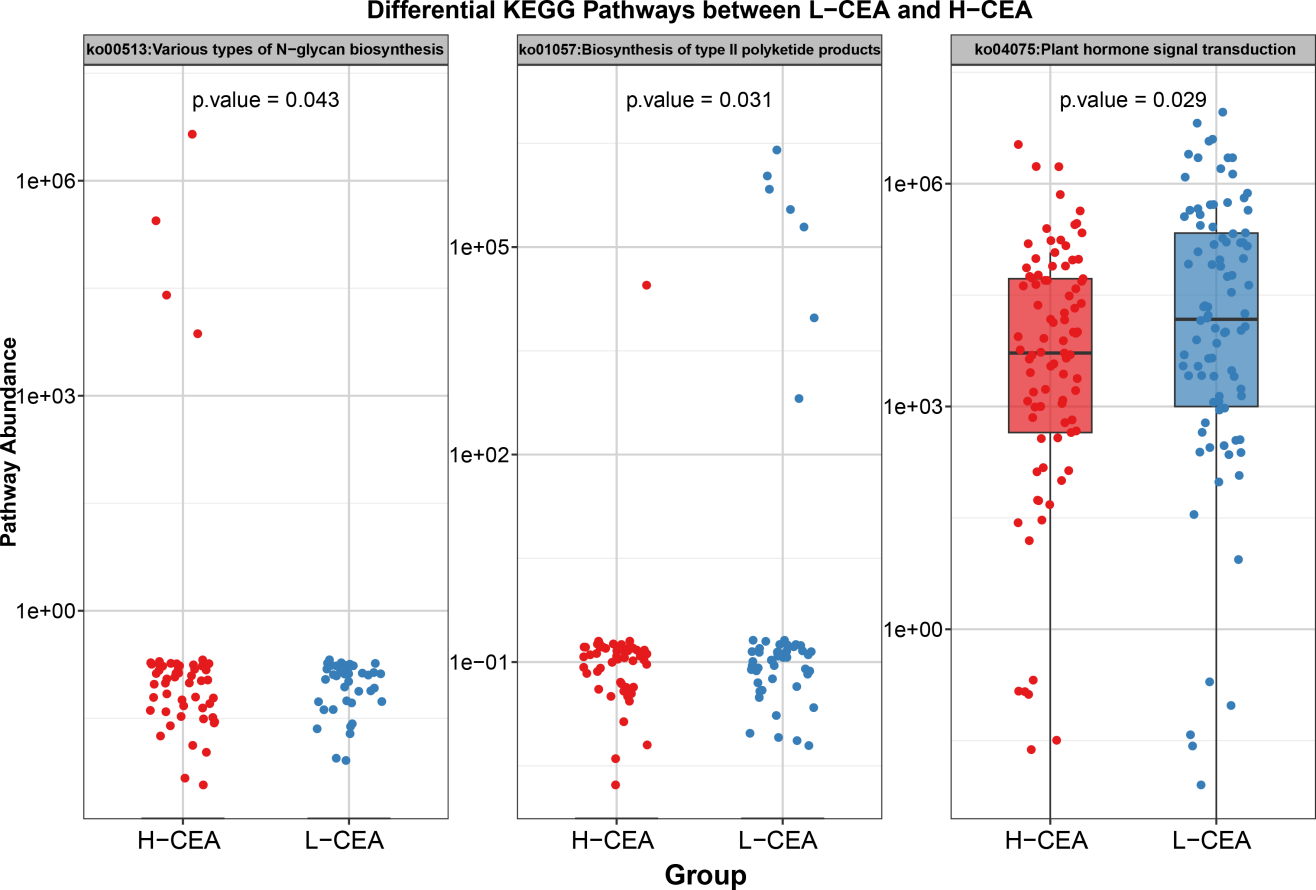
Differential KEGG pathway predictions based on gut microbiota between L-CEA and H-CEA groups. Bars are color-coded by group (L-CEA: blue; H-CEA: red), with the *y*-axis listing pathway descriptions and the *x*-axis showing log10-scaled pathway abundance values. Pathway predictions were generated using PICRUSt2 based on 16S rRNA gene sequencing data, with statistical significance determined by LEfSe at the *P* < 0.05 threshold.

To investigate the functional differences of gut microbiota under different CEA levels in CRC patients, this study used PICRUSt 2 to perform predictive analysis of microbiota function based on 16S rRNA sequencing data and focused on specific metabolic pathways related to disease progression. A total of 177 KEGG pathways were identified, three of which were significantly different between the H-CEA and L-CEA groups (*P* < 0. 05). Figure 3 and Table S2 demonstrated the abundance distribution of these three key pathways. The results showed that there was one pathway in the H-CEA group with significantly higher abundance, namely, ko00513: Various types of N-glycan biosynthesis (*P* = 0. 043), and there were two pathways in the L-CEA group with significantly higher abundance than that in the H-CEA group, namely, ko01057: Biosynthesis of type II polyketide products (*P* = 0. 031) and ko04075: Plant hormone signal transduction (*P* = 0. 029). These differential pathways are potentially related to CEA levels, and their upregulation in the corresponding groups may reflect the characteristic changes in metabolic functions of the gut microbiota under different CEA levels, suggesting that there are significant functional differences in specific metabolic pathways between the two groups.

### Correlation of CEA-associated gut microbiota with tumor-infiltrating immune cells

**Fig 4:**
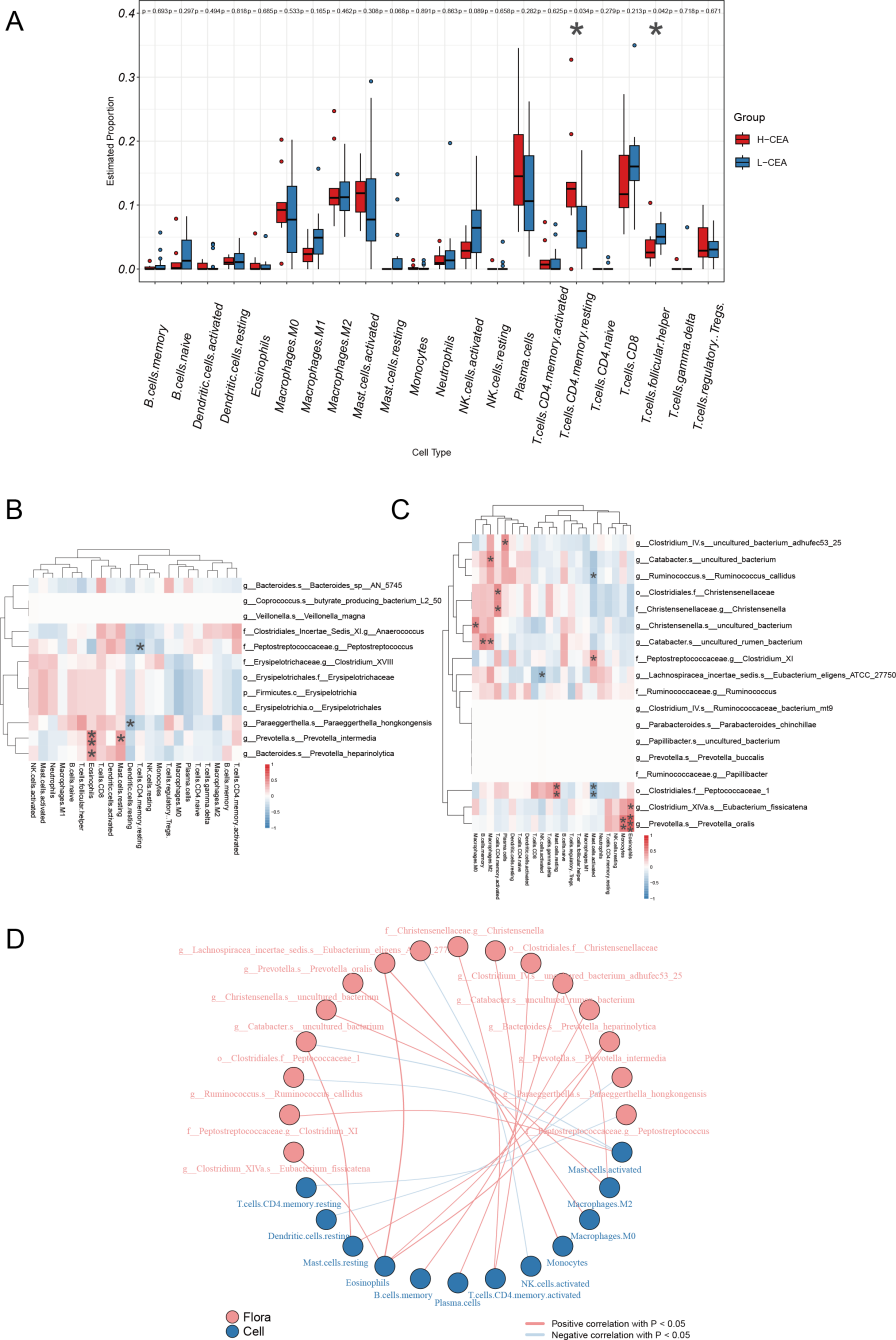
Relationship between CEA-associated gut microbiota and infiltrating immune cells in CRC (**A**) Box plot of the estimated proportion of immune cells between L-CEA and H-CEA groups. Box-and-whisker plots depicting estimated proportions of 22 immune cell types in CRC patients stratified by H-CEA (red) and L-CEA (blue) status. *x*-axis: immune cell types; *y*-axis: relative abundance. Statistical significance: *P < 0.05; **P < 0.01 (Wilcoxon test). (**B**) Heat maps of correlation between dominant colonies in L-CEA and tumor-infiltrating immune cells. (**C**) Heat maps of correlation between dominant colonies in H-CEA and tumor-infiltrating immune cells. *x*-axis: immune cells; *y*-axis: bacterial taxa. Color gradient: red (positive correlation, *r* > 0) to blue (negative correlation, *r* < 0); intensity scales with |*r*|. The depth of the color indicates the size of the Pearson correlation coefficient. The "*" in the graph represents the size of the *P*-value: no * for *P*-value ≥ 0.05, * for 0.01 ≤ *P* < .05, ** for 0.001 ≤ *P* < .01, and *** for *P* < 0.001. (**D**) Network diagram of correlations between gut microbiota and immune cell differences associated with CEA levels. Nodes in different colors in the figure represent gut microbiota and immune cells, and the connection lines between nodes indicate a significant correlation between nodes. The blue line indicates that the Spearman correlation coefficient is less than 0 (negative correlation), while the red line indicates that the Spearman correlation coefficient is greater than 0 (positive correlation).

Tumor-infiltrating immune cells play a key role in the tumor immune micro-environment and have a significant impact on tumor-associated immune responses, both inhibiting tumor growth and potentially promoting tumor metastasis and immune escape (17), making them a potential target for tumor immunotherapy. Using transcriptome sequencing data, we conducted an in-depth study of 22 infiltrating immune cell compositions in 25 CRC patients, covering the assessment of the differences in immune cell abundance, the correlation analysis of colonies and immune cells, and the construction of association networks. The box line plots in Fig. 4A demonstrated the abundance differences of different immune cell types in the H-CEA and L-CEA groups, in which the abundance of resting memory CD4 T cells was higher in the H-CEA group than in the L-CEA group (*P* = 0. 034), and the abundance of T follicular helper cells was higher in the L-CEA group than in the H-CEA group (*P* = 0. 042). Figure 4B and C demonstrate the correlation between significantly elevated microbiota and immune cells in the L-CEA and H-CEA groups, respectively. We found that *g__Ruminococcus. s__Ruminococcus_callidus* was significantly positively correlated with activated mast cells in the H-CEA group. While in the L-CEA group, *g__Prevotella. s__Prevotella_intermedia* was significantly positively correlated with eosinophils. Figure 4D further demonstrated the association between bacterial groups and immune cells. These results indicated that there were significant differences in immune cell infiltration between the H-CEA and L-CEA groups and that CEA-associated dominant gut microbiota were significantly associated with a variety of tumor-infiltrating immune cells in CRC patients, suggesting that CEA-associated differential gut microbiota have a potential modulatory role in shaping the immune micro-environment in CRC.

### CEA-associated differential gut microbiota and immune-related genes

**Fig 5:**
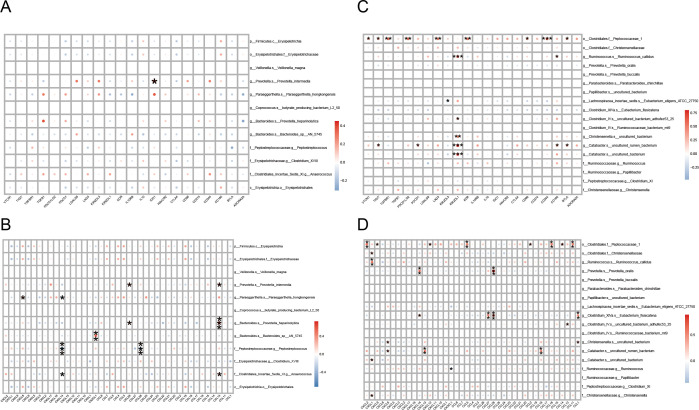
Correlation between CEA-related differences in gut microbiota and immune-related genes. (**A**) Heat map of correlation between the dominant microbiota and immunosuppressive genes in the L-CEA group. (**B**) Heat map of the correlation between the dominant microbiota and chemokines in the L-CEA group. (**C**) Heat map of the correlation between the dominant microbiota and immunosuppressive genes in L-CEA. (**D**) Heat map of the correlation between the dominant microbiota and chemokines in H-CEA. In the figure, the horizontal coordinate is the immune-related genes, and the vertical coordinate is the bacteria. The red indicates the positive correlation, and the blue indicates the negative correlation. The color depth indicates the size of the Pearson correlation coefficient, and the color from light to dark indicates the value of the phase relationship from small to large. The "*" in the graph represents the size of the *P*-value: no * for *P*-value ≥ 0.05, * for 0.01 ≤ *P* < .05, ** for 0.001 ≤ *P* < .01, and *** for *P* < 0.001.

We conducted an in-depth analysis of the correlation between CEA-associated differential gut microbiota and common immune-related genes, revealing a potential link between CEA-associated gut microbiota and host immunity. As shown in Fig. 5, among L-CEA-associated differential bacteria, *g__Prevotella.s__Prevotella_intermedia* was associated with immunosuppressive genes IDO1 (Fig. 5A), chemokines (CCL13 and CCL28) (Fig. 5B), immune checkpoints (IDO1 and IDO2) (Fig. S1A), immune-activating genes KLRC1 (Fig. S1B), and chemokine receptor (CXCR1) (Fig. S1C) were significantly positively correlated. Among the differential bacteria in the H-CEA group, *g__Ruminococcus. s__Ruminococcus_callidus* was positively correlated with immunosuppressive genes (KIR2DL1 and CD160) (Fig. 5C), chemokine (CXCL-1) (Fig. 5D), immune checkpoints (CD27, CD160, etc.) (Fig. S1D), immune-activating genes (TNFRSF17, TNFRSF13B, etc.) (Fig. S1E), and chemokine receptor (CCR3) (Fig. S1F) were significantly positively correlated. Given the critical role of the human immune system in tumorigenesis and progression, these findings suggest that CEA-associated differential gut microbiota may have an impact on the expression of immune-related genes.

### Identification of CEA-associated differential biological functional pathways and their correlation with differential microbiota

**Fig 6:**
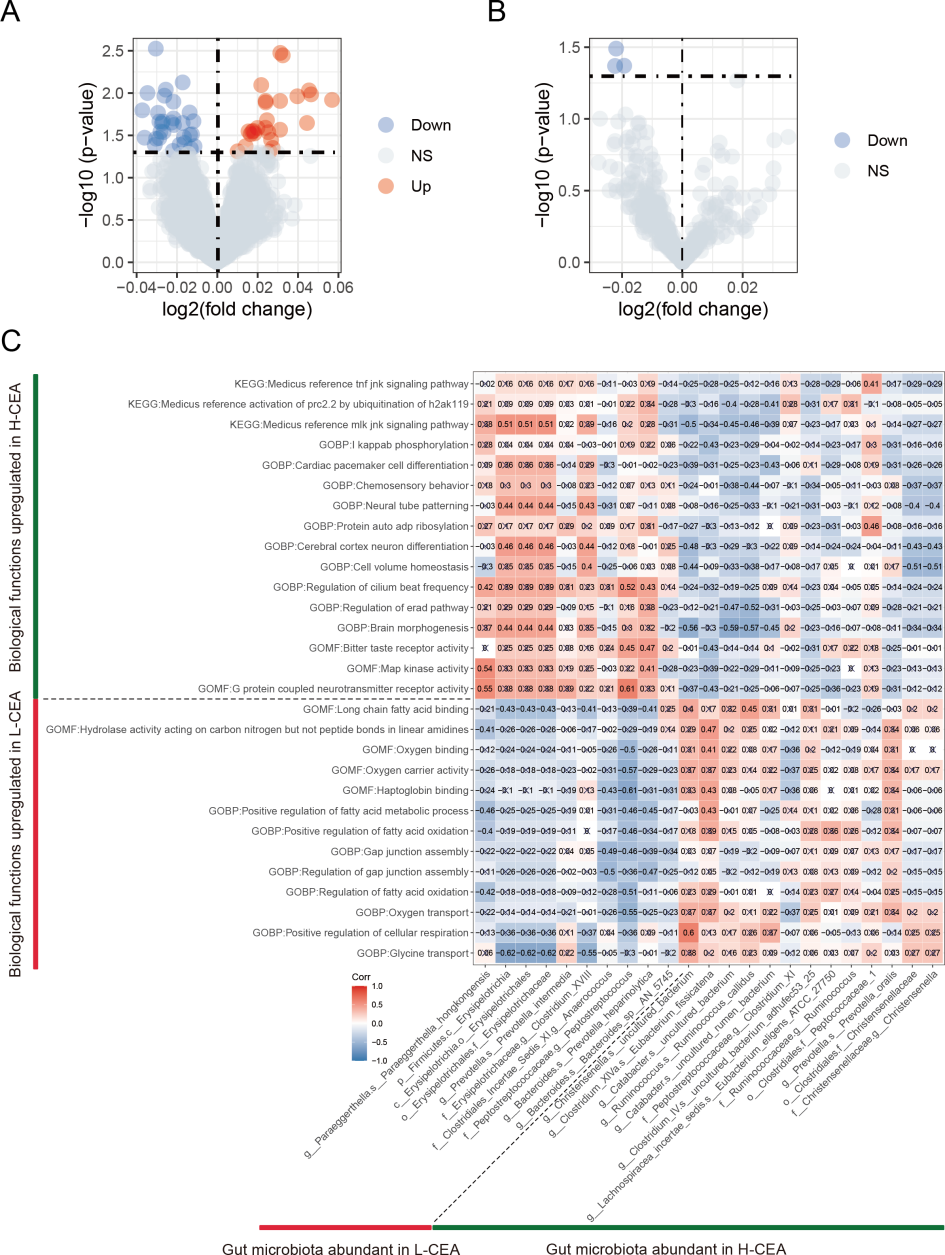
Identification of CEA-related differential pathways and their correlation with CEA-related differential gut microbiota. (**A**) GO items volcano plot of the associated differential expression in the L-CEA versus H-CEA group. (**B**) KEGG pathways volcano plot of the associated differential expression in the L-CEA versus H-CEA group. The horizontal coordinates indicate log 2 (fold change). The farther the point is from the center, the larger the multiple of the difference; The vertical coordinates represent −log 10 (*P*-value). The closer the point is to the top, the more significant the difference in expression. Each dot represents the differentially expressed gene detected. Red indicates upregulated genes. Blue indicates downregulated genes. Gray indicates no differential genes. (**C**) CEA-related difference correlation graph of gut microbiota with CEA-related differences in GOBP, GOMF, and KEGG pathways. The horizontal coordinate of the graph is bacteria. The vertical coordinates are the GOBP, GOMF, and KEGG pathways. In this figure, red indicates a positive correlation, blue indicates a negative correlation, color depth and numerical value indicate the size of Spearman correlation coefficient, and color from light to dark indicates the value of the phase relationship from small to large. The symbol "×" in the figure represents the size of the *P*-value: the presence of "×" means the *P*-value ≥ 0.05, and the absence of "×" means the *P*-value＜0.05.

To identify the differential biological functional pathways in the L-CEA and H-CEA groups and the correlation of different gut microbes with these pathways, we performed a comprehensive analysis of tumor tissue samples from 25 CRC patients and converted the gene expression matrix and gut microbial species abundance matrix into scoring matrices using the ssGSEA method. These matrices were then analyzed by KEGG and GO analysis, which includes cellular components (CC), molecular functions (MF), and biological processes (BP). Subsequently, by analyzing the GO and KEGG pathway scoring matrices of the two groups differently (Fig. 6A and B), in the L-CEA group, we identified 26 significantly upregulated GO pathways (e.g., GOBP: positive regulation of fatty acid beta oxidation [logFC = 0. 057, *P* = 0. 012] and GOMF: haptoglobin binding [logFC = 0. 032, *P* = 0. 003]). There was no significant KEGG pathway. In the H-CEA group, we identified a total of 31 significantly upregulated GO pathways (e.g., GOBP: celluar lipid biosynthesis process [logFC = 0. 023, *P* = 0. 022] and GOMF: phosphate ion binding [logFC = 0. 037, *P* = 0. 016]) and three significantly upregulated KEGG pathways (e.g. KEGG: medicus reference TNF JNK signaling pathway [logFC = 0. 022, *P* = 0. 043]). Complete information on GO and KEGG-enriched entries is provided in Table S3. These findings highlight the different biological functions.

Further correlation analyses revealed significant associations of specific gut microbes with biological pathways (see Fig. 6C; Table S4 for results). For example, in the H-CEA group, there was a statistically significant positive correlation between *g__Ruminococcus. s__Ruminococcus_callidus* and GOMF: Long-chain fatty acid binding (*r* = 0. 45, *P* = 0. 023). In the L-CEA group, there was a statistically significant negative correlation between *s__Paraeggerthella_hongkongensis* and GOMF: G protein couple neurotransmitter receptor activity (*r* = 0. 55, *P* = 0. 005). These findings suggest that CEA-associated differential gut microbiota may have an impact on tumor progression in patients by participating in specific biological functional pathways.

### Modeling the prediction of H-CEA and L-CEA using differential gut microbiota characterization

LEfSe analysis (Fig. 2B) identified 30 CEA-related differential gut microbiota features. Based on the results of the importance ranking of gut microbiota features (Fig. S2), we retained all 30 features for the construction of a prediction model of CEA levels based on RF and XGBoost algorithms. The gut microbiota data of 187 CRC patients containing CEA labels included in the study were randomly divided into a training set (70%) and a validation set (30%).

**Fig 7:**
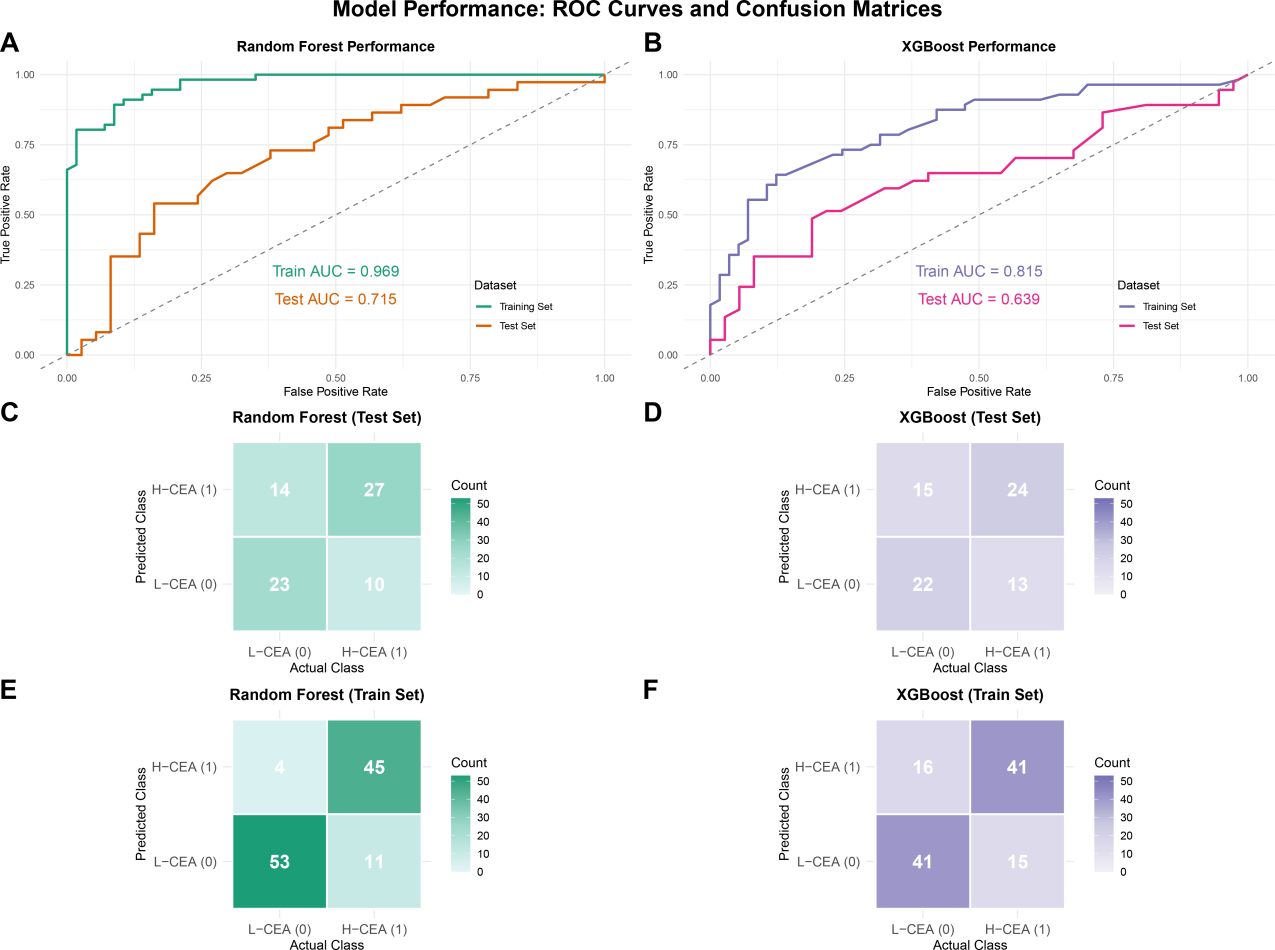
Machine learning models based on CEA-associated differential gut microbiota to predict serum CEA levels in CRC. (**A**) ROC curves of the RF model training set and testing set. (**C**) Confusion matrix in the training set of the RF model. (**E**) Confusion matrix in the RF model testing set. (**B**) ROC curves of the XGBoost model training set and testing set. (**D**) Confusion matrix in the training set of the XGBoost model. (**F**) Confusion matrix in the XGBoost model testing set. The AUC values in the figures represent AUC values, with higher AUC values indicating better model prediction performance. The horizontal axis represents the model’s prediction label, while the vertical axis represents the true sample status. The values "1" and "0" signify positive and negative predictions, respectively. The numbers within different boxes represent the sample count. The color depth is proportional to the number of samples; the greater number of samples, the darker the color depth.

In the CEA prediction model based on the RF algorithm, the AUC value of the training cohort was as high as 0.969 (Fig. 7A), while the AUC value of the test cohort was 0.715, which was lower than that of the training set but still higher than the randomized guessing level of 0.5, indicating that the RF model has some clinical applications in predicting CEA levels. The confusion matrix of the training cohort (Fig. 7C) showed that the number of true negative (TN) and true positive (TP) samples was significantly higher than that of false negative (FN) and false positive (FP) samples, indicating that the model had a high classification accuracy and stability in the training set and was able to effectively differentiate between CEA-positive and -negative samples. In the test cohort (Fig. 7E), the number of TN and TP samples still exceeds that of FN and FP samples despite a slight decrease in the model’s performance, indicating that the RF model still maintains a certain level of generalization ability and reliability.

For the XGBoost-based CEA prediction model, the ROC curve analysis shows that the AUC value of the XGBoost training cohort is 0.815 (Fig. 7B), and that of the test cohort is 0.639, which suggests that the XGBoost model may not be as good as the RF model in terms of generalization ability and stability and that its performance in practical applications may be more dependent on the quality of the data and feature selection. The confusion matrix of the training cohort (Fig. 7D) shows a similar trend, with significantly more TN and TP samples than FN and FP samples. In the test cohort (Fig. 7F), TN and TP samples still outnumber FN and FP samples.

Both models showed potential to predict CEA levels. In comparison, the RF model performs better in balancing the training and validation performance. The XGBoost model, although it performs moderately well in the training set, its performance in the test set decreases significantly, suggesting a tendency of overfitting. Therefore, under the current data set conditions, the RF model is more advantageous in distinguishing H-CEA from L-CEA patients and is more suitable for this classification task.

## DISCUSSION

16S rRNA sequencing is commonly used to analyze the composition and diversity of microbial communities, while transcriptomic sequencing focuses on the gene expression of specific cells or tissues in a particular state, and these two sequencing technologies are widely used in microbiology and genetic research. In this study, the integration of these two methods not only identified the differences in the gut microbiota of CRC patients with different CEA levels but also revealed the mechanism of the interaction between the gut microbiota and the tumor immune micro-environment, which effectively fills the gap in the study of the mechanism of the CEA levels and the progression of CRC.

The gut microbiota plays a key role in inflammatory and malignant gastrointestinal diseases (18). LEfSe analysis showed that 30 bacteria at different taxonomic levels were associated with CEA levels and 18 bacteria at different taxonomic levels were associated with H-CEA. One of the highest LDA-scoring bacteria was the genus *Ruminococcus* in the family *Ruminococcaceae*, and precise to the species level, we identified one of them, *Ruminococcus.callidus*, which phylogenetic analyses confirmed to be part of an evolutionarily conserved branch of the genus *Ruminococcus*, with the same taxonomic level of *R. flavefaciens*, *R. albus,* and *R. bromii*, distinguishing it from other species clusters such as *R. gnavus*.^19^ Several studies have revealed the important role of *R. callidus* in diseases. While previous studies have linked *R. callidus* to obesity-associated CRC risk (20) and immune checkpoint inhibitor therapy (21), our study is the first to identify its specific and significant enrichment in CRC patients with high serum CEA levels. This association was further corroborated by its high ranking at machine learning feature importance, suggesting its potential as a novel fecal biomarker for elevated CEA in CRC. The strain also shows a unique pattern of enrichment in a variety of other diseases. For example, *R. callidus* was identified by a machine learning algorithm as a beneficial strain for treatment responders in breast cancer CDK4/6 inhibitor treatment (22) and correlated with cognitive scores in children (23), suggesting neurodevelopmental effects. Hepatic encephalopathy studies, on the other hand, found an increased abundance of the phenylalanine decarboxylase gene in *R. callidus*, which was associated with accumulation of the neurotoxin phenylethylamine and neurological damage (24). In summary, *R. callidus* presents a complex pattern of disease association, and its specific mechanisms of action (e.g., specific metabolites and immune interactions) still need to be explored in depth, which is crucial for understanding the pathogenic mechanisms of gut microbiota and developing intervention strategies. CEA has been found to correlate with harmful bacteria in the gut of CRC patients (9). In this study, *R. callidus* was found to be significantly enriched in the H-CEA group. These findings established a direct link between a specific gut microbiota member and a clinically significant tumor marker, moving beyond general associations with CRC risk or therapy response.

Predictive analysis of gut microbiota function based on PICRUSt2 showed that the N-glycan biosynthetic pathway was significantly enriched in patients in the H-CEA group. As a key component of glycoconjugates (25), N-glycans play an important role in the development, diagnostic prognosis, and treatment of CRC, and their aberrant glycosylation has become an important area of research. Large-scale plasma N-glycomics studies have confirmed that the serum levels of specific glycan structures are significantly elevated in CRC patients compared to healthy controls and colorectal adenoma patients and further upregulated in advanced cases (26). Inhibition of the IFNγ signaling pathway promotes an immunosuppressive micro-environment (27), which in turn mediates immune escape, which may be one of the mechanisms of its malignant pro-cancer properties. This experiment also showed that removal of such pro-tumorigenic branched N-glycans exposed immunogenic mannose structures, enhanced the recognition ability of DC-SIGN-positive immune cells, and effectively activated the antitumor immune response. In addition, N-glycosylation of the tight junction protein claudin-3 enhances paracellular permeability and promotes a malignant phenotype, whereas inhibition of N-glycan synthesis significantly reduces claudin-3 expression levels (28). For therapeutic applications, inhibitors targeting N-glycan biosynthesis were effective in inhibiting malignant behaviors such as clone formation, migration, and invasion of CRC cells *in vitro* experiments (29). Although aberrant N-glycosylation is a well-established hallmark in CRC progression and prognosis, our study uniquely links this pathway to the gut microbiota functional profile specifically associated with high serum CEA levels. The enrichment predicted by PICRUSt2 suggests that the gut microbiota in H-CEA patients may contribute to or reflect the dysregulated N-glycan biosynthesis observed in advanced CRC, potentially explaining part of the association between high CEA and worse prognosis. Future multicenter large-sample studies are needed to further validate the generalizability of this mechanism and assess the potential group selection bias.

Gene expression data from the two groups of patients were obtained by transcriptomic sequencing, and the relative abundance of 22 immune cells was calculated by back-convolution using the CIBERSORT algorithm. The analysis showed that there were different immune infiltrating cells between the two groups: significantly higher abundance of resting memory CD4 T cells in the H-CEA group and higher abundance of T cells follicular helper in the L-CEA group. Studies have shown that CD4 T cells, especially CD4 memory T cells, are critical for immunotherapy-induced tumor regression (30). The higher the infiltration of activated CD4 memory T cells in tumor tissues, the better the prognosis of cancer patients (31); in contrast, infiltration of resting memory CD4 T cells is associated with poor prognosis, and the abundance of these two cell subsets is significantly negatively correlated. Several studies of diagnostic gene markers for CRC have also found (32–34) that resting memory CD4 T cells are significantly associated with the expression of oncogenes. However, by stratifying patients based on serum CEA levels, we provide a novel immunological correlate for this clinically relevant biomarker. The contrasting abundance of T follicular helper cells in the L-CEA group further underscores the distinct immune micro-environment associated with different CEA levels.

Microbial communities within the tumor micro-environment (TME) interact with immune cells to significantly influence mucosal immune responses, with microbes regulating T cell differentiation and expansion. In this study, we found that the abundance of *R. callidus* was positively correlated with mast cell (MC) infiltration and upregulation of chemokine CXCL1 expression. Peritumoral hyperinfiltration of MCs is an independent predictor of poor prognosis in CRC liver metastases (35) and is significantly and positively correlated with lymph node metastasis and clinical stage (36). In cancer progression, MCs act mainly by promoting angiogenesis and lymphangiogenesis. Under hypoxia and acidosis conditions, MCs release pro-angiogenic mediators such as VEGF-A/VEGF-A/CXCL8/endothelin-1 and VEGF-C/D pro-lymphangiogenic factors (37–39) to rebuild the tumor vasculature network and accelerate the metastatic progression, which may be associated with stem cell factors (SCF). Tumor-derived SCF binds to the c-Kit receptor on the MC surface (40), triggering degranulation to release VEGF/PDGF/FGF-2 (41), as well as activation of the β-catenin pathway and stimulation of protease expression to enhance the supportive effect on colon cancer cells. In contrast, pharmacological blockade of c-Kit inhibits this pathway and retards tumor growth (42). Several tumor-derived chemokines, including CXCL1, which significantly promotes tumor angiogenesis, induce migration toward cancer foci by activating MC surface receptors (43). Notably, MC function is dynamically regulated by tumor micro-environmental signals: IL-1, IL-4, IL-6, and TNF-α activate antitumor effects, whereas VEGF, matrix metalloproteinase, trypsin-like enzymes, and IL-10 promote malignant progression (44). In addition, MCs can affect the differentiation of CD4 T cells. Bacteria and fungi can directly activate MCs, inducing their degranulation and release of VEGF and inflammatory factors (45), while certain micro-organisms can also inhibit FcεRI-mediated MC function. In inflammatory bowel disease, MC intestinal accumulation and increased permeability due to dysbiosis form a vicious cycle (46), but the specific mechanism of MC-microbial crosstalk in the pathogenesis of CRC remains to be analyzed. The present study suggests that *R. callidus* may promote tumor angiogenesis and lymphangiogenesis by upregulating MC infiltration and CXCL1 expression to promote CRC progression, a mechanism that coincides with the clinical characteristics of high CEA patients with highly invasive and metastatic tumors.

By constructing a heat map of the association between gut microbiota and the GO/KEGG pathway, the analysis of biological pathway differences between the two groups of patients revealed that the abundance of *R. callidus* was significantly and positively correlated with GOMF: Long-chain fatty acid binding. Long-chain fatty acids (LCFAs) are the main components of dietary fatty acids, including palmitate, oleate, stearate, linoleate, and linolenate. The current study points out that LCFAs are involved in tumor progression through multiple mechanisms. For example, palmitic acid is catalyzed by long-chain lipoyl coenzyme A synthetase 1 (ACSL1) to generate palmitoyl coenzyme A, which disrupts circadian homeostasis through ZDHHC5-mediated palmitoylation of the CLOCK protein, forming an ACSL1-CLOCK positive feedback loop (47), which promotes metabolic disorders and tumorigenesis. In addition, LCFA-CoA can directly inhibit the metastasis suppressor gene NME1, weakening its ability to regulate epithelial-mesenchymal transition (EMT) and stromal protein hydrolysis (48, 49), thus accelerating the metastasis of high-fat diet-associated breast cancer. LCFAs can also activate the AMPK to regulate the phosphorylation cascade of MNK-eIF4E (50), and its oxidative metabolism (FAO) not only provides energy but also builds a pro-metastatic lipid micro-environment that promotes lymphatic metastasis in particular (51). Of particular note, the molecular mechanisms of LCFAs in intestinal microbiota-macrophage interactions and their clinical significance on colorectal cancer (CRC) progression have been systematically elucidated (52). The positive correlation between *R. callidus* and LCFA binding activity suggests a potential novel metabolic interface between this gut bacterium and CRC progression in high-CEA patients. Despite the detrimental roles of LCFAs and their metabolism in CRC, the specific contribution of *R. callidus* to LCFA-mediated metabolic reprogramming in the context of high CEA is a new finding.

ML, as a core branch of artificial intelligence, has become increasingly valuable in the field of tumor diagnosis and treatment management. ML-based methods are able to integrate multidimensional features to construct predictive models for accurate prediction of disease classification labels and continuous variables, which has significant potential for non-invasive marker identification in cancer. RF and XGBoost algorithms have been widely used in the study of blood index prediction in CRC patients due to their powerful feature correlation analysis (53). Existing results have confirmed that such algorithms can construct an integrated learning system based on serum biomarkers to effectively predict CRC disease risk and TNM staging (54). Although CEA is often included as a predictive model feature, there is a gap in studies using gut microbiota to predict serum CEA levels. In this study, we constructed RF and XGBoost models to predict serum CEA levels in CRC patients by screening differential microbiota characteristics. The results of the training set showed that both models had excellent performance (AUC >0. 90), while the RF model in the test set showed more stable generalization ability (AUC: 0. 715 vs 0. 639). While ML models incorporating serum biomarkers for CRC diagnosis or staging exist, and gut microbiota signatures have been used to predict CRC presence or response, predicting a continuous blood biomarker level directly from fecal microbiota composition represents a significant novel approach. This non-invasive strategy holds promise for monitoring CEA dynamics and potentially stratifying patients based on microbiota-associated CEA levels.

Although this study provides new insights into the differential identification of gut microbiota in serum CEA levels in CRC patients, it must be acknowledged that it has several limitations that need to be urgently improved at the level of methodological design, study dimensions, and mechanism exploration. First, although there were 187 samples for the fecal microbiome analysis, the tumor tissue for transcriptome analysis was from only 25 patients. The smaller sample size may not be sufficient to capture the full range of true transcriptomic differences, reducing statistical efficacy and increasing the risk of false-positive or false-negative results. Second, there are obvious limitations in the spatial resolution of the study perspective. The analysis of intra-tumoral microbiota and its comparison with adjacent normal mucosa is crucial, the latter revealing the interaction between microbiota translocation and the tumor immune micro-environment. The lack of simultaneous sampling from multiple sites (feces-tumor-paraneoplastic mucosa) prevented us from resolving the colonization dynamics of the microbiota from the intestinal lumen to the tumor ecological niche, which constitutes an important gap in the depth of microbiome studies. More importantly, the causal chain of the current findings has not yet been established, and *in vitro* experiments and animal models are needed to verify the interactions between microbiota and CEA expression. In the future, we will strive to break through these limitations and systematically analyze the mediating mechanism of gut microbiota metabolites in CEA release through metabolomics combined with transcriptomics so as to reveal the causal network of the microbiota-metabolism-immunity axis that drives cancer progression.

### Conclusion

In this study, we identified 30 gut microbial species significantly associated with serum CEA concentrations in a CRC cohort, among which *R. callidus* showed specific enrichment in the high-CEA group. This bacterium may act as a key driver to promote the invasive phenotype and metastatic progression of CRC through activation of mast cell infiltration, up-regulation of chemokine CXCL1 expression, and reprogramming of long-chain fatty acid metabolic pathways. The RF and XGBoost prediction model constructed based on CEA-associated bacterial markers further confirmed its potential for translational application in the noninvasive assessment of CEA levels in CRC patients.

## MATERIALS AND METHODS

### Subject information and sample collection

The study was approved by the Ethics Committee of Guangxi Medical University Cancer Hospital, and all subjects signed an informed consent form. Stool samples were collected from 236 CRC patients between January and December 2021, of which 198 were quality-controlled by 16S rRNA sequencing. Based on clinicopathological data (gender, age, TNM stage, tumor volume, microsatellite status, etc.) and a median CEA of 3.54 ng/mL, 187 patients were classified into a high CEA group (H-CEA, *n* = 93) and a low CEA group (L-CEA, *n* = 94). Another 25 of these postoperative tumor tissues ((H-CEA, *n* = 8; L-CEA, *n* = 17) were collected for transcriptomic sequencing.

Inclusion criteria for subjects: (i) colorectal adenocarcinoma diagnosed by surgical pathology or colonoscopic biopsy with tumor location, TNM staging (AJCC 8th edition), and degree of differentiation documented; (ii) no antitumor therapy, such as surgery, chemotherapy, or radiotherapy, prior to sample collection; (iii) no previous history of intestinal surgery; (iv) no history of other malignancies; (v) no use of antibiotics or microecological agents in the month prior to sample collection; (vi) cognitively normal preparations; (vii) normal cognitive function.

Stool samples were collected on the day of admission, and patients were instructed to retain mid-stool in a sterile collection tube (to avoid urine contamination). After dispensing 200 mg into Eppendorf (EP) tubes, the samples were placed at −80°C for freezing (55). Tumor tissues were collected on the day of surgery, and fresh tumor tissues of 3–5 mm in diameter were collected from multiple points and placed in liquid nitrogen for quick-freezing and preservation within 30 min (56).

### 16S rRNA sequencing and gut microbiota analysis

For DNA extraction, a 200 mg stool sample was weighed and mixed with Tris-EDTA buffer using the MOBIO PowerSoil DNA Extraction Kit, a procedure designed to continuously optimize the efficiency and quality of DNA extraction (57). After the extraction was completed, the samples with high-quality DNA were strictly selected for PCR amplification, a critical step that builds up the foundation for the accuracy of subsequent analysis. In the PCR amplification stage, we selected specific primers 341F (5′-CCTACGGGGNGGCWGCAG-3′) and 805R (5′-GACTACHVGGGGTATCTAATCC-3′), which can accurately target the V3 and V4 regions of the 16S rRNA gene, and selectively amplify the target fragments by PCR (58). The amplified products were then characterized by 2% agarose gel electrophoresis, and we focused on bands in the 300–350 bp range to ensure the accuracy and specificity of the amplified fragments. The concentration of the PCR products was determined using the Quant-iT PicoGreen dsDNA Assay Kit, and all samples were mixed equimolarly. After mixing, the samples were quantified using the KAPA Library Quantification Kit KK4824, a delicate process designed to ensure consistency and stability of the samples prior to sequencing.

On the Illumina PE250 platform, we performed high-throughput sequencing of qualified libraries using 2 × 250 bp chemistry. After sequencing, we harvested the raw sequencing data in FASTQ format. In order to ensure the quality of the data, we used QIIME2 (59) (Quantitative Insights Into Microbial Ecology version 2) to perform a series of processes, including quality control and denoising, species annotation, and low abundance and contaminant filtering, which successfully eliminated the low-quality sequences and retained the high-quality data for the subsequent analysis. We have successfully eliminated low-quality sequences and retained high-quality data for subsequent analysis.

For microbial community analysis, we utilized the Greengene database v13.8 for detailed annotation of the gut microbiota. Extraction and analysis of amplicon sequence variants (ASVs)/operational taxonomic units (OTUs) were performed in the phyloseq package (version 1. 26.1). In assessing microbial diversity, we used α-indices of diversity, including Chao1, ACE, coverage, observed, Shannon, and Simpson indices, which accurately characterize the number and uniformity of microbial species within a single sample. The β-diversity (Bray-Curtis and Jaccard distance) was used to compare differences in microbial community structure between samples, revealing similarities and differences between samples. These analyses were realized with the help of ADONIS and ANOSIM analyses in the vegan package v2.5.6.

In order to deeply mine the classification and comparison information in the high-dimensional data, we applied the "mixOmics" package v6.6.2 to implement partial least squares discriminant analysis (PLS-DA). At the same time, LEfSe (60) was used to set a strict threshold of LDA score | LDA score | >2 and *P*-value < 0. 05 and accurately screened for gut microbiota that could significantly differentiate the two groups of CRC patients. The impacts of these gut microbiota were assessed by the LDA score and presented as a visual bar graph using the ggplot2 package v3.4.0.

For the screened key gut microbiota, we further predicted the potential enrichment of KEGG pathway between the two groups of CRC patients based on the 16S rRNA sequencing data with the help of Phylogenetic investigations of Communities by Reconstruction of Unobserved States II (61) (PICRUSt2) software v2.3.0. potential enrichment of KEGG pathway between the two groups of CRC patients. We used the nonparametric Mann-Whitney test to compare the differences in α-diversity indices between the two groups and analyzed the degree of KEGG pathway enrichment between the two groups in depth with the help of the vegan package v2.5.6. All data analyses were efficiently performed in R software v4.3.2, and the statistical significance criterion was set at a *P* value of <0.05 (two-tailed test).

### Transcriptomic sequencing and tumor immune micro-environment analysis

In this study, RNA was extracted from 25 CRC tumor tissue samples using the Trizol Total RNA Extraction Kit, covering 17 patients in the L-CEA group and 8 patients in the H-CEA group. The extracted RNA was subjected to a stringent quality control process whereby integrity was verified by electrophoresis and purity was accurately assessed with the aid of a micro-UV spectrophotometer to ensure the quality of the RNA for subsequent analysis. After removal of rRNA interferences, cDNA libraries were carefully prepared according to the detailed instructions of the RNA-seq Sample Preparation Kit (VAHTS Stranded mRNA-seq Library Prep Kit for Illumina). These transcriptomic libraries were then sequenced on the state-of-the-art Illumina NovaSeq 6000 System, generating approximately 6G of data per sample. Upon completion of sequencing, the quality of the sequencing data was initially assessed using FastQC software to ensure high quality and accuracy. Then, the HISAT2 tool was used to accurately align the sequences with the reference genome to lay the foundation for gene expression analysis. Finally, with the help of StringTie software and the established gene models, the gene expression levels were quantified and the expression abundance of each gene was calculated in transcripts per million (TPM), which provided the key gene expression data for the subsequent studies.

CIBERSORT is a state-of-the-art algorithm focused on precisely quantifying immune cell composition from RNA sequencing data. It cleverly utilizes gene expression features specific to different immune cells and accurately identifies and classifies gene expression profiles with the help of machine learning algorithms (62). In this study, we applied the CIBERSORT R script to combine known reference gene expression profiles with the gene expression data of the composite samples to be analyzed and constructed a model using support vector regression. With this model, we successfully transformed the TPM matrix obtained from transcriptome sequencing into a matrix representing the relative proportions of 22 different immune cell types and their functional states. Further, we deeply integrated the microbial composition matrix with the immune cell proportion matrix and calculated the correlation coefficients of each pair of columns in the merged matrix by using the rcorr function in R, thus revealing the potential correlation between immune cells and microbial composition.

### Functional enrichment analysis of CEA-related transcriptome sequencing

On the R software v4.3.2 platform, we performed an in-depth functional enrichment analysis of RNA sequencing data. The Single Sample Gene Set Enrichment Analysis (ssGSEA) algorithm was applied to gene sets in GMT format (downloaded from the GSEA website (https://www.gsea-msigdb.org/gsea/index.jsp), including c2. cp. kegg. v2024.1. Hs. symbols. gmt, and c5. go. v2024.1. Hs. symbols. Gmt were applied. ssGSEA algorithms were computed for each gene set in each sample based on a descending ordering of gene expression levels with corresponding ssGSEA scores. These scores were able to quantify the extent to which members of a particular gene set were coordinately up or downregulated in the sample. In this way, we were able to assess the overall expression activity of each gene set in the samples, thereby laying the foundation for further functional analysis. To generate the gene set scoring matrices, we used the "ssgsea" function in the "GSVA" package v1. 46.0. This process translates the gene set enrichment into numerical scores for subsequent analysis. Next, we used the L-CEA group as a control group and analyzed the differences in GO items and KEGG pathways between the two groups using the "limma" algorithm integrated in the "TCGAbiolinks" package v2. 25.3. During the screening process, we set strict conditions: *P*-value < 0. 05 and |log2FC| > 0 to ensure that the screened set of differential genes was statistically significant and biologically relevant. The GO items cover three levels: biological process (BP), molecular function (MF), and cellular component (CC), which provided us with a comprehensive view of gene function. In order to visualize these significantly different GO projects and KEGG pathways, we plotted volcano maps with the help of the powerful plotting capabilities of the "ggplot2" package v3.4.0.

### Machine learning model building and identification of gut microbial markers

To predict serum CEA levels in CRC patients based on gut microbiome features, two ML architectures, RF and XGBoost, were designed and implemented in this study. While traditional statistical models are difficult to resolve nonlinear relationships in high-dimensional data, ML methods can more effectively capture complex data patterns due to fewer assumption constraints (63), thus demonstrating superior prediction performance. RF is an integrated ML algorithm based on decision trees. Its core mechanism lies in constructing multiple decision trees and aggregating (e.g., majority voting or mean computation) their predictions to significantly improve the overall accuracy and robustness of the model (64). RF has become one of the most widely used tools in the field of data mining and machine learning due to its excellent performance. One of the key advantages of the algorithm is its built-in feature importance assessment mechanism, which can efficiently rank variables (65), and is particularly suitable for analyzing high-dimensional and complex data structures. Currently, RF can be conveniently implemented by the open-source R language package “randomForest”(66). XGBoost is an efficient gradient boosting tree algorithm, which is good at dealing with high-dimensional complex data (67). The algorithm improves prediction accuracy by iteratively constructing decision trees, with each new tree focusing on correcting the residuals of the prior model. Its regularization effectively prevents overfitting and enhances model robustness by limiting the number of leaf nodes and weight size (68). Combined with Shapley additive explanations (SHAP), XGBoost demonstrates high accuracy in predicting disease incidence and prevalence trends (68). XGBoost can also be implemented by the open-source R language package “xgboost.” More and more microbiomics studies are skillfully applying machine learning techniques to accurately differentiate between health and disease states or to target key features for predicting clinical outcomes (11).

### Statistical methods

In R software version 4.3. 2, we analyzed categorical data using the Pearson χ test and continuous data related to clinical baseline characteristics using the *t*-test, which allowed for a comprehensive statistical evaluation of the data. Pearson correlation analysis was performed with the “Hmisc” package version 5.1. 1 to investigate the complex association between gut microbiota, immune cell counts, and immune-related gene expression (11). At the same time, Spearman correlation analysis was performed with the “ggcorrplot” package version 0.1.4. 1 as a way to analyze the correlation between the two groups of dominant microbiotas and the enriched molecular functions (MF), biological processes (BP), and KEGG pathway. For the visual presentation of the analysis results, we used a variety of tools: the “pheatmap” package version 1.0. 13 was utilized to draw heatmaps, the “ggcorrplot” package version 0.1.4. 1 to draw correlation plots, the “ggplot2” package version 3.5. 2 was used to draw violin plots, box-and-line plots, volcano plots, and machine-learning display plots, the “Igraph” package version 2.1. 4 was used to construct network diagrams, while “Cytoscape software” version 3. 10. 1 was used for complex network analysis.

## Data Availability

The original contributions presented in the study are included in the article material. The data that support the findings of this study are openly available in the National Genomics Data Center (NGDC) database at https://www.cncb.ac.cn/. The accession number for 16S rRNA sequencing is HRA012812, and the accession number for transcriptome sequencing is HRA012776.

## SUPPLEMENTAL MATERIAL

Fig. S1 msphere.00454-25-s0001.tif Heat map of correlation between dominant bacteria and immune checkpoints, immune-activating genes, and chemokine receptors in H-CEA and L-CEA.

Fig. S2 msphere.00454-25-s0002.tif Importance of RF and XGBoost model bar graph.

Supplemental material msphere.00454-25-s0003.docx Legends for supplemental figures and tables.

Table S1 msphere.00454-25-s0004.docx Results of LEfSe analysis.

Table S2 msphere.00454-25-s0005.docx KEGG pathways in the gut microbiota of CRC patients in H-CEA and L-CEA group.

Table S3 msphere.00454-25-s0006.xlsx List of differential GO items and KEGG pathways of H-CEA and L-CEA groups.

Table S4 msphere.00454-25-s0007.xlsx Association between CEA-associated GO and KEGG enrichment and the dominant gut microbiota in H-CEA and L-CEA groups.

ASM does not own the copyrights to Supplemental Material that may be linked to, or accessed through, an article. The authors have granted ASM a non-exclusive, world-wide license to publish the Supplemental Material files. Please contact the corresponding author directly for reuse.
