# Supplementary figures and images for: Identification and predictive machine learning model construction of gut microbiota associated with carcinoembryonic antigens in colorectal cancer
Source: mSphere. 2025 Sep 17;10(10):e00454-25. doi: 10.1128/msphere.00454-25 (PMC12570507; doi:10.1128/msphere.00454-25)

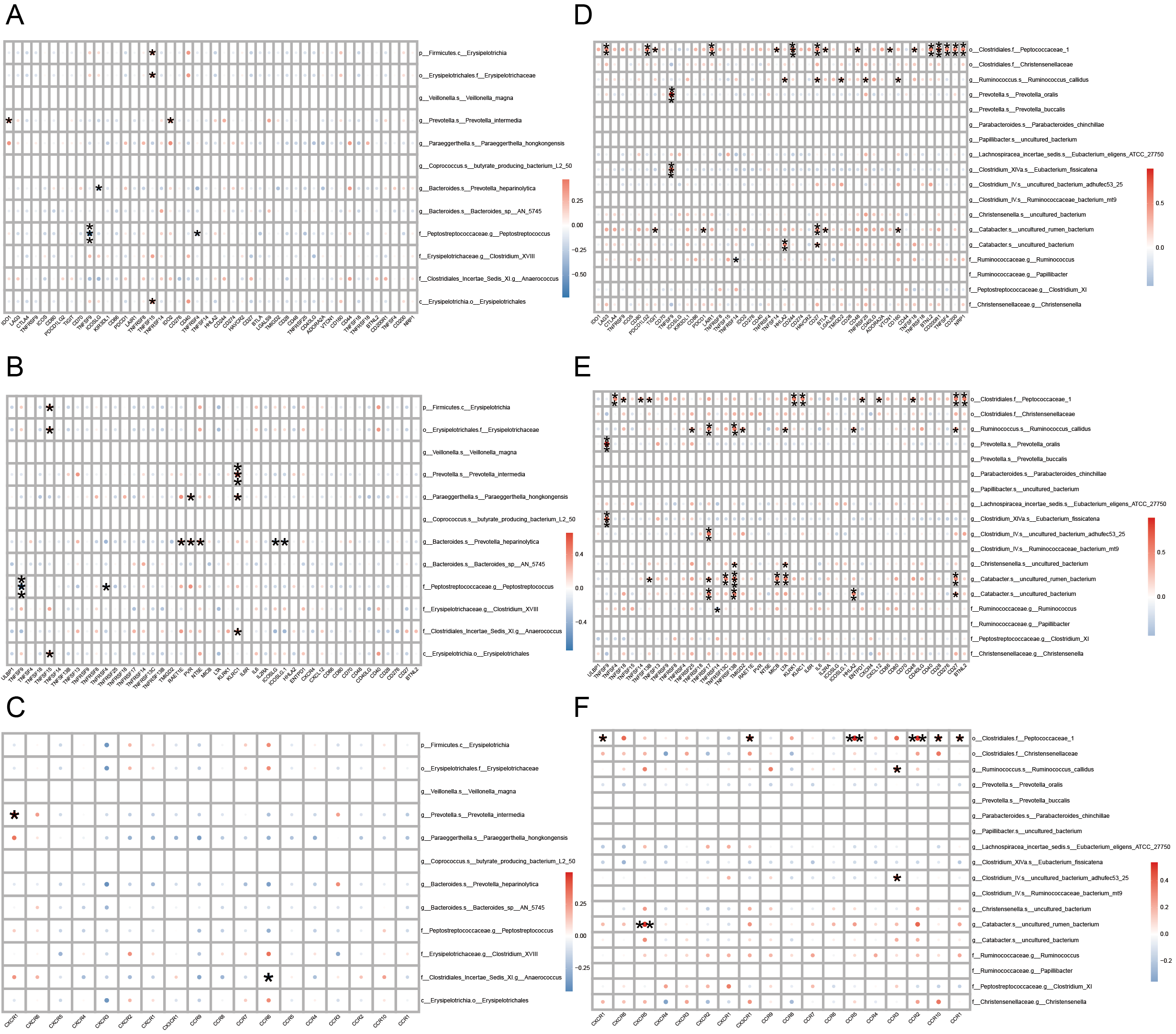

Supplement: Fig. S1 — Heat map of correlation between dominant bacteria and immune checkpoints, immune-activating genes, and chemokine receptors in H-CEA and L-CEA. [file msphere.00454-25-s0001.tif]

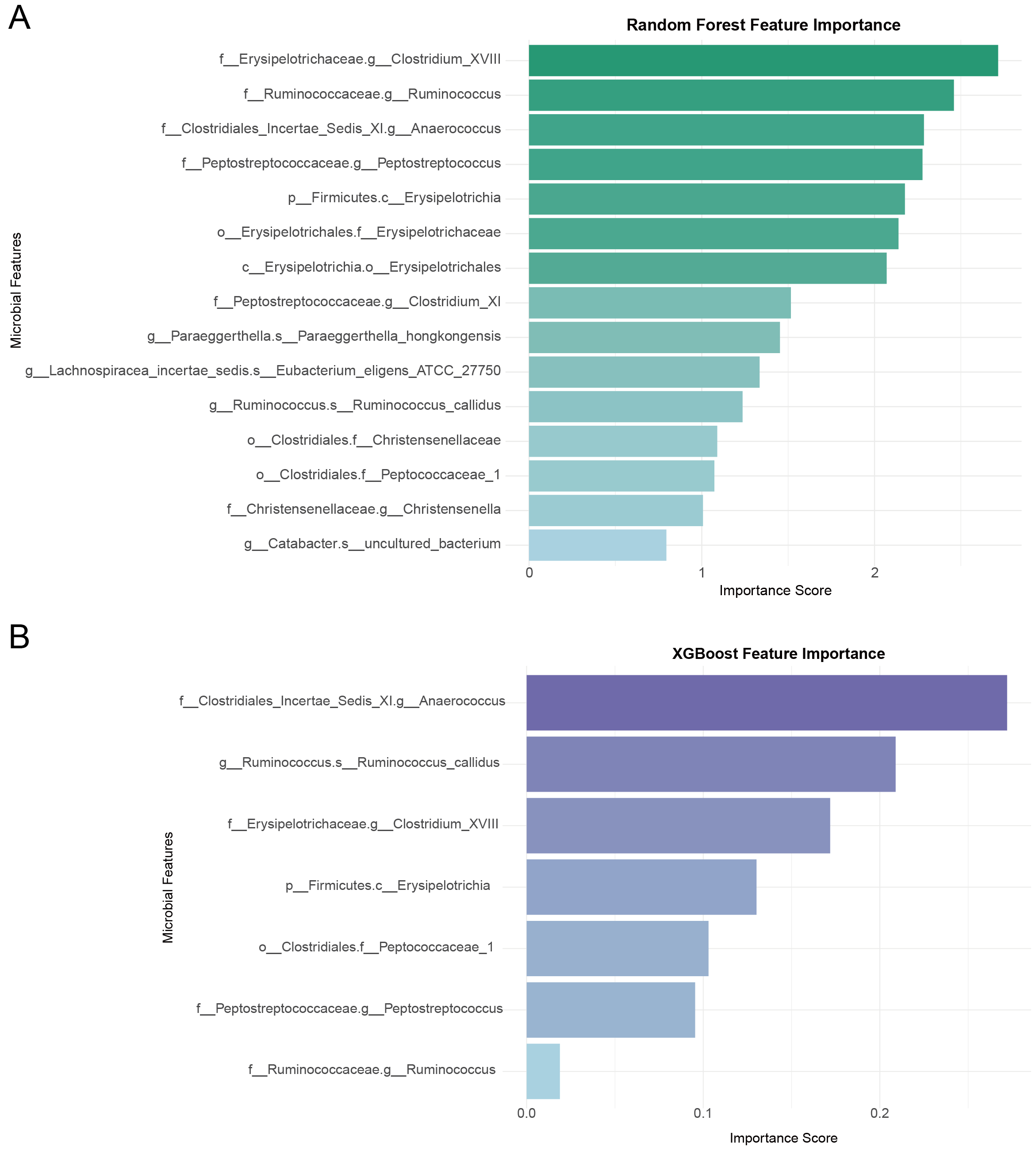

Supplement: Fig. S2 — Importance of RF and XGBoost model bar graph. [file msphere.00454-25-s0002.tif]
