# Supplementary material for: Identification and predictive machine learning model construction of gut microbiota associated with carcinoembryonic antigens in colorectal cancer
Source: mSphere. 2025 Sep 17;10(10):e00454-25. doi: 10.1128/msphere.00454-25 (PMC12570507; doi:10.1128/msphere.00454-25)
Supplement: Supplemental material — Legends for supplemental figures and tables. [file msphere.00454-25-s0003.docx]

**Supplementary Figures**

**Fig.S1A. Heat map of correlation between dominant bacteria and immune checkpoints in L-CEA.**

**Fig.S1B. Heat map of correlation between dominant bacteria and immune-activating genes in L-CEA.**

**Fig.S1C. Heat map of correlation between dominant bacteria and chemokine receptors in L-CEA.**

**Fig.S1D. Heat map of correlation between dominant bacteria and immune checkpoints in H-CEA.**

**Fig.S1E. Heat map of correlation between dominant bacteria and immune-activating genes in H-CEA.**

**Fig.S1F. Heat map of correlation between dominant bacteria and chemokine receptors in H-CEA.**

Horizontal coordinate is gene, vertical coordinate is colony, red represents positive correlation, blue represents negative correlation, color depth represents Pearson correlation coefficient size, color from light to dark indicates Pearson correlation coefficient value from small to large**.** The "*" in the graph represents the size of the p-value: No * for P-value ≥ 0.05, * for 0.01 ≤ P<0.05, ** for 0.001 ≤ P<0.01, *** for P<0.001.

**Fig.S2A. Importance of RF model bar graph.**

**Fig.S2A. Importance of XGBoost model bar graph.**

The horizontal axis represents the feature importance score, with larger values indicating a higher contribution of the feature to the model classification. The vertical axis lists the key flora taxonomic units, covering different levels of classification from phylum to species. The horizontal axis of the graph displays the score that quantifies the importance of the feature; the higher the score, the greater the importance of the feature.

**Supplementary Tables**

**Table.S1. Results of LEfSe analysis.**

Taxonomy: CEA-related gut microbiota information; Group: group with significant abundance of differential species; LDA: effect value of CEA-associated gut microbiota after log10 treatment; species with LDA scores (log10) greater than 2 and p-values less than 0.05 are shown in Table.

**Table.S2.** **KEGG pathways in the gut microbiota of CRC patients in H-CEA and L-CEA group.**

KEGG_id: description: KEGG pathway; Mean in H-CEA: the predicted abundance value of this pathway in each sample in H-CEA; Mean in L-CEA: the predicted abundance value of this pathway in each sample in L-CEA.

**Table.S3. List of differential GO items and KEGG pathways of H-CEA and L-CEA groups.**

GO items: Enriched GO entries. KEGG pathways: Enriched KEGG entries. LogFC: FC represents the folding change, that is, the ratio of the expression of H-CEA and L-CEA. The logarithm is taken as the base of 2. Statistically significant when p-value is less than 0.05.

**Table.S4. Association between CEA-associated GO and KEGG enrichment and the dominant gut microbiota in H-CEA and L-CEA groups.**

The r.value is the Spearman correlation coefficient value. P-value less than 0.05 is statistically significant.
