# Supplementary material for: Identification and predictive machine learning model construction of gut microbiota associated with carcinoembryonic antigens in colorectal cancer
Source: mSphere. 2025 Sep 17;10(10):e00454-25. doi: 10.1128/msphere.00454-25 (PMC12570507; doi:10.1128/msphere.00454-25)
Supplement: Table S1 — Results of LEfSe analysis. [file msphere.00454-25-s0004.docx]

**Table.S1. Results of LEfSe analysis**

| Taxonomy | Group | LDA | Pvalue |
| --- | --- | --- | --- |
| f__Ruminococcaceae.g__Ruminococcus | H-CEA | 4.935 | 0.03298 |
| f__Peptostreptococcaceae.g__Clostridium_XI | H-CEA | 3.825 | 0.02839 |
| g__Lachnospiracea_incertae_sedis.s__Eubacterium_eligens_ATCC_27750 | H-CEA | 3.674 | 0.03174 |
| o__Clostridiales.f__Christensenellaceae | H-CEA | 3.556 | 0.04447 |
| f__Christensenellaceae.g__Christensenella | H-CEA | 3.552 | 0.04447 |
| o__Clostridiales.f__Peptococcaceae_1 | H-CEA | 3.497 | 0.03691 |
| g__Ruminococcus.s__Ruminococcus_callidus | H-CEA | 3.286 | 0.00687 |
| g__Clostridium_IV.s__Ruminococcaceae_bacterium_mt9 | H-CEA | 2.962 | 0.04266 |
| g__Prevotella.s__Prevotella_oralis | H-CEA | 2.942 | 0.04266 |
| g__Catabacter.s__uncultured_bacterium | H-CEA | 2.611 | 0.00473 |
| g__Clostridium_IV.s__uncultured_bacterium_adhufec53_25 | H-CEA | 2.597 | 0.02862 |
| g__Parabacteroides.s__Parabacteroides_chinchillae | H-CEA | 2.509 | 0.02558 |
| g__Christensenella.s__uncultured_bacterium | H-CEA | 2.339 | 0.00212 |
| g__Catabacter.s__uncultured_rumen_bacterium | H-CEA | 2.312 | 0.02129 |
| g__Papillibacter.s__uncultured_bacterium | H-CEA | 2.221 | 0.02479 |
| g__Clostridium_XlVa.s__Eubacterium_fissicatena | H-CEA | 2.208 | 0.00376 |
| f__Ruminococcaceae.g__Papillibacter | H-CEA | 2.202 | 0.02479 |
| g__Prevotella.s__Prevotella_buccalis | H-CEA | 2.124 | 0.01256 |
| f__Peptostreptococcaceae.g__Peptostreptococcus | L-CEA | 4.847 | 0.03121 |
| p__Firmicutes.c__Erysipelotrichia | L-CEA | 4.729 | 0.01134 |
| c__Erysipelotrichia.o__Erysipelotrichales | L-CEA | 4.729 | 0.01134 |
| o__Erysipelotrichales.f__Erysipelotrichaceae | L-CEA | 4.729 | 0.01134 |
| f__Erysipelotrichaceae.g__Clostridium_XVIII | L-CEA | 4.384 | 0.02625 |
| g__Prevotella.s__Prevotella_intermedia | L-CEA | 3.668 | 0.02442 |
| g__Coprococcus.s__butyrate_producing_bacterium_L2_50 | L-CEA | 3.613 | 0.03154 |
| f__Clostridiales_Incertae_Sedis_XI.g__Anaerococcus | L-CEA | 3.486 | 0.02724 |
| g__Bacteroides.s__Prevotella_heparinolytica | L-CEA | 3.483 | 0.04492 |
| g__Paraeggerthella.s__Paraeggerthella_hongkongensis | L-CEA | 3.443 | 0.00406 |
| g__Veillonella.s__Veillonella_magna | L-CEA | 3.364 | 0.04492 |
| g__Bacteroides.s__Bacteroides_sp__AN_5745 | L-CEA | 2.779 | 0.04492 |

Footnote: Taxonomy: information of CEA-associated gut microbiota; Group: CRC patients with H-CEA and L-CEA; LDA (log10): effect value of CEA-associated gut microbiota, which was after log10 transformation; the species showed in the table were selected by the threshold of |LDA score| >2 and P < 0.05. P value: P<0.05 as statistical significance.
