# Supplementary material for: Identification and predictive machine learning model construction of gut microbiota associated with carcinoembryonic antigens in colorectal cancer
Source: mSphere. 2025 Sep 17;10(10):e00454-25. doi: 10.1128/msphere.00454-25 (PMC12570507; doi:10.1128/msphere.00454-25)
Supplement: Table S2 — KEGG pathways in the gut microbiota of CRC patients in H-CEA and L-CEA group. [file msphere.00454-25-s0005.docx]

**Table.S2. KEGG pathways prediction in the gut microbiota of CRC patients in H-CEA and L-CEA group.**

| KEGG_id:description | Mean.In.L-CEA | Mean.In.H-CEA | Pvalue |
| --- | --- | --- | --- |
| ko04075: Plant hormone signal transduction | 476758.5939 | 118449.9189 | 0.02872 |
| ko01057: Biosynthesis of type II polyketide products | 51470.69947 | 302.3225806 | 0.03124 |
| ko00513: Various types of N-glycan biosynthesis | 0 | 51301.51333 | 0.04322 |

KEGG_id: description: KEGG pathway; Mean in H-CEA: the predicted abundance value of this pathway in each sample in H-CEA; Mean in L-CEA: the predicted abundance value of this pathway in each sample in L-CEA.
